# Hypoplasia of the Atlas Does Not Correlate With Subaxial Canal Stenosis: A Retrospective Cohort Study

**DOI:** 10.7759/cureus.109541

**Published:** 2026-05-24

**Authors:** Noriaki Yokogawa, Kevin Yoon, Linus Lee, Jonathan P Japa, Mark Ehioghae, Abdi Khalif, Zion M Rouege, Banuja Munasinghe, Addisu Mesfin

**Affiliations:** 1 Department of Orthopaedic Surgery, Kanazawa University, Kanazawa, JPN; 2 Department of Orthopaedic Surgery, MedStar Washington Hospital Center, Washington, D.C., USA; 3 Department of Orthopaedic Surgery, MedStar Health Research Institute, Washington, D.C., USA

**Keywords:** atlas hypoplasia, cervical stenosis, congenital deformity, ct, prevalence, spine surgery

## Abstract

Background and objective

It remains unclear whether atlas hypoplasia is causative of symptomatic cervical canal stenosis or represents an independent and unrelated finding. There have been few dedicated studies describing atlas hypoplasia, and the existing literature has focused almost entirely on Asian populations. Therefore, the aims of the current study were threefold. First, we aimed to characterize the differences in cervical foraminal sagittal measurements by race and sex. Second, we evaluated the impact of age on cervical sagittal diameter and assessed the relationship between atlas hypoplasia and subaxial canal stenosis. Finally, we investigated the relationship between dens size and canal stenosis.

Methods

This was a single-institution retrospective cohort study approved by the Institutional Review Board at the University of Rochester School of Medicine and Dentistry. An overarching patient cohort was created by selecting patients with a Current Procedural Terminology (CPT) code of 72125-7 (CT of the cervical spine) and an International Classification of Diseases, 9th Edition (ICD-9) code 723.0 (cervical myelopathy) between January 1, 2014, and December 31, 2014. A total of 851 patients fulfilled the inclusion criteria. Variables collected included demographic and radiographic data. The inner sagittal diameters (ISD) at every cervical spine level, including the atlas, were measured. The prevalence of atlas hypoplasia was compared by age group. The correlation between each ISD was calculated. Among cases of atlas hypoplasia, the proportion of patients with subaxial canal stenosis was investigated.

Results

A total of 851 patients were included in the analysis. The mean ISD of the atlas was 31.4 ± 2.2 mm, and we defined atlas hypoplasia as an atlas ISD ≤ 27.0 mm. The mean space available for the cord (SAC) of the atlas was 19.0 ± 2.0 mm (range: 12.8-27.1). There was no significant difference in either the ISD of the atlas or the prevalence of atlas hypoplasia between the age groups. Pearson’s correlation analysis showed that the ISD of the atlas had a strong correlation with the SAC of the atlas (r = 0.86, p < 0.01); a moderate correlation with both the sagittal diameter of the dens (coefficient = 0.48, p < 0.01) and the ISD of C2 (r = 0.59, p < 0.01) to C3 (r = 0.44, p < 0.01); and a weak correlation with the ISD of C4 (r = 0.37, p < 0.01), C5 (r = 0.38, p < 0.01), C6 (r = 0.36, p < 0.01), and C7 (r = 0.37, p < 0.01). A sub-cohort analysis of the 17 cases of atlas hypoplasia revealed that the mean ISD of the atlas was 26.3 ± 0.7 mm, and the mean SAC of the atlas was 14.5 ± 0.8 mm within this cohort. Regarding the rate of canal stenosis, the atlantal SAC was less than 14 mm in 17.6% of the cases, and the ISD of C2 to C7 was < 12 mm in 0%, 35.3%, 41.2%, 64.7%, 52.9%, and 47.1% of the cases, respectively.

Conclusions

The prevalence of atlas hypoplasia was not correlated with older age, which differs from subaxial developmental canal stenosis. Furthermore, although there was no strong correlation between the ISD of the atlas and each subaxial ISD, cases with atlas hypoplasia often involved subaxial canal stenosis.

## Introduction

The dimensions of the upper cervical canal were first studied by Steel in 1968 [1]. He proposed the “Rule of Thirds,” in which the odontoid process, spinal cord, and cerebrospinal fluid each occupy one-third of the canal diameter at the level of the atlas [1]. Developmental stenosis can contribute to cervical spondylotic myelopathy and traumatic spinal cord injury [2-4]. The cervical spinal canal is widest at the level of the atlas and narrows caudally from C2 to C5 [3,5]. Cervical spondylotic myelopathies most commonly occur at these caudal levels [6]. However, rare cases, primarily anecdotal or consisting of small case series from Japanese and East Asian populations, have described symptomatic cervical stenosis secondary to atlas hypoplasia [7-11].

Morphologically, atlas dysplasia is categorized as either partial atlas agenesis or a hypoplastic but complete ring. Hypoplasia is defined as a sagittal canal diameter at least one standard deviation below the mean. It is thought to result from the dysfunction of two of the three atlas ossification centers [12]. Proposed mechanisms include impaired dorsal extension of the lateral ossification centers due to abnormal chondrogenesis or premature fusion and early ossification of the neural arches [9,10,13]. Because atlas hypoplasia stems from a different pathophysiologic mechanism than subaxial developmental stenosis, it may require distinct approaches to screening and management. It remains unclear whether atlas hypoplasia is causative of symptomatic cervical canal stenosis or represents an independent and unrelated finding [13-15].

There have been few dedicated studies describing atlas hypoplasia, and the existing literature has focused almost entirely on Asian patient populations [5]. Therefore, the aims of the current study were threefold. First, we aimed to characterize the differences in cervical foraminal sagittal measurements by race and sex. Second, we evaluated the impact of age on cervical sagittal diameter and assessed the relationship between atlas hypoplasia and subaxial canal stenosis. Finally, we investigated the relationship between dens size and canal stenosis.

## Materials and methods

Data source

This was a single-institution retrospective cohort study approved by the Institutional Review Board at the University of Rochester School of Medicine and Dentistry. An overarching patient cohort was created by selecting patients with a Current Procedural Terminology (CPT) code of 72125-7 (CT of the cervical spine) and an International Classification of Diseases, 9th Edition (ICD-9) code 723.0 (cervical myelopathy) between January 1, 2014, and December 31, 2014 [16,17]. Only patients with complete CT cervical spine series, including axial and sagittal reconstructions, were included in this study. Cases were excluded if they met any of the following criteria: poor image quality, non-adult patients, history of or current spinal trauma, history of cervical spine surgery, congenital subaxial spinal deformities, such as os odontoideum and Klippel-Feil syndrome, or a history of ossification of the posterior longitudinal ligament. If multiple cervical spine CT scans for the same case were identified, only the initial CT scan was included. All images were reviewed using the institution’s picture archiving and communication system (PACS) server. A total of 851 patients fulfilled the inclusion criteria. Variables collected included demographic and radiographic data.

Radiographic measurement

**Figure 1:**
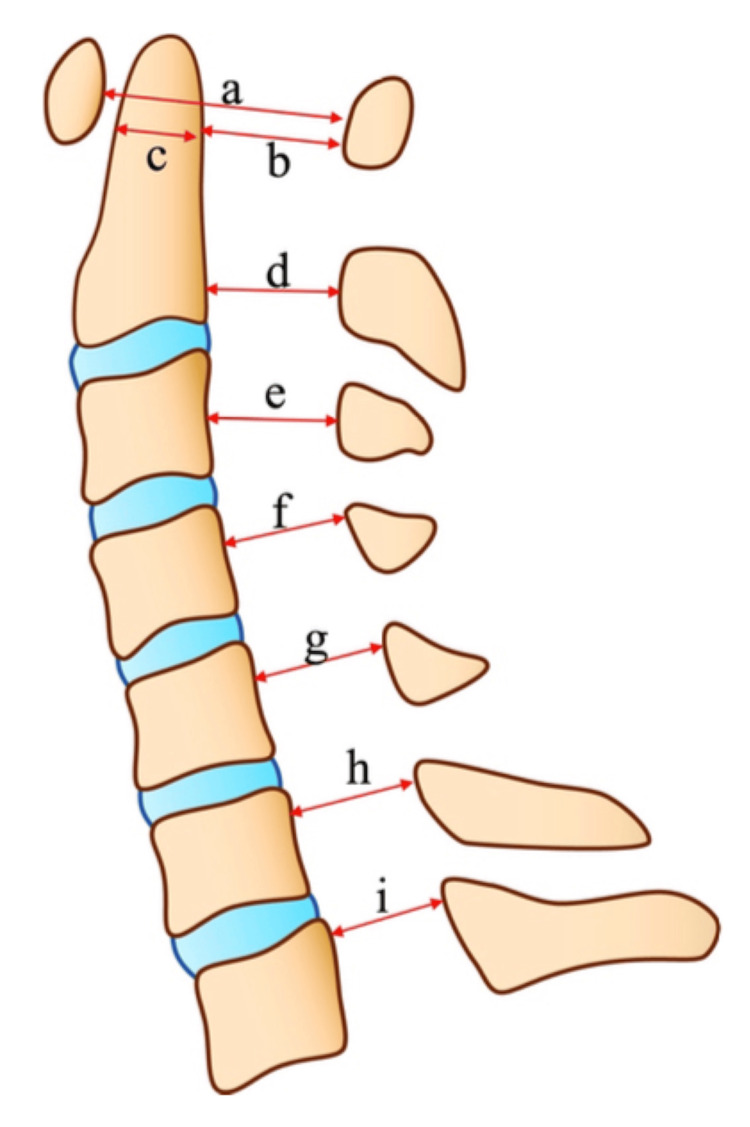
Sagittal measurements The figure is an author-drawn illustration of how sagittal measurements were performed. Line a represents how the calculation of the inner sagittal diameter (ISD) of the atlas was performed, measuring the longest point between the anterior and posterior arches facing the spinal canal. Line b represents how the space available for the spinal cord (SAC) was measured from the most posterior aspect of the dens to the most anterior portion of the posterior arch of the atlas. Line c represents how the sagittal diameter of the dens was measured, using the largest portion found within the atlanto-axial junction. Line d-i represents how the measurement of the inner-sagittal distance for the subsequent sub-axial cervical levels was performed Image credit: authors

Cases were divided into those with a discontinuous atlas (atlas agenesis) and those with a complete ring of the atlas. All sagittal images were oriented as shown in the illustration in Figure 1. The following parameters were measured for those with complete rings: the inner sagittal diameter (ISD) of the atlas, the space available for the cord (SAC) at the level of the atlas, the sagittal diameter of the dens, and the ISD of every subsequent level of the cervical spine. For each sagittal parameter, the maximum diameter was selected using the axial and sagittal CT images. Figure 1 depicts how measurements were made on a CT scan. 

Data Analysis

Radiographic data were presented as a mean and standard deviation (SD), and compared by sex, race, and age to evaluate demographic variation. Hypoplasia of the atlas was defined as an ISD less than one standard deviation below the cohort mean, as described in prior literature [10]. Subgroup analysis was performed for cases categorized by atlas hypoplasia to examine the proportion of cases with an atlas SAC less than 14 mm or a subaxial ISD less than 12 mm - both of which are standardized measurements of canal stenosis [18,19]. Statistical analyses performed to compare the groups included the following: independent t-test for continuous variables, one-way ANOVA for variables with three or more degrees of freedom, and chi-squared test for categorical variables. Pearson’s correlation coefficient was calculated for each radiographic parameter in relation to the demographic variables listed above. Statistical significance was defined as p < 0.05. All statistical analyses were performed using SPSS Statistics software Version 27 (IBM Corp., Armonk, NY).

## Results

A total of 851 patients met the inclusion criteria, 803 of whom had demographic data available. There were 24 (2.9%) cases of atlas agenesis, of which 23 (2.8%) had a posterior defect, and one (0.1%) had an anterior defect. In the remaining 803 cases, the mean ISD of the atlas was 31.4 ± 2.2 mm (range: 24.5-38.4), and the mean SAC of the atlas was 19.0 ± 2.0 mm (range: 12.8-27.1). Within the study’s cohort, atlas hypoplasia was defined as an ISD ≤ 27.0 mm. The average ISD was greatest at C2, at 16.2 ± 1.5 mm (range: 11.2-20.6), and stabilized between C4-C7, with values ranging from 13.6 to 13.7 mm on average. The mean sagittal diameter of the dens was 11.3 ± 1.0 mm (range: 8.6-14.5), occupying 36.2 ± 2.9% (range: 27.7-50.0%) of the ISD. All parameters were significantly higher in males than in females. Caucasians also had significantly larger ISDs than African Americans at all cervical levels. Caucasians also had significantly larger ISDs than all other races included in the study, except at the C7 level. Table 1 presents the sagittal diameter measurements at each cervical level, stratified by sex and race, for the 803 patients with complete demographic data.

**Table 1:** Sagittal diameter at each level according to sex and race ^*^Statistically significant difference between Caucasians and African Americans or others, respectively. ^**^Statistically significant difference between Caucasians and African Americans The values are given as the mean and standard deviation. P-values were calculated using an independent t-test for continuous variables, one-way ANOVA for variables with three or more degrees of freedom, and chi-squared test for categorical variables ISD: inner sagittal diameter; SAC: space available for the cord; SD: sagittal diameter

Variable	C1 ISD (mm)	C1 SAC (mm)	Dens SD (mm)	C2 ISD (mm)	C3 ISD (mm)	C4 ISD (mm)	C5 ISD (mm)	C6 ISD (mm)	C7 ISD (mm)
Overall (n = 803), mean ± SD	31.4 ± 2.2	19.0 ± 2.0	11.3 ± 1.0	16.2 ± 1.5	14.1 ± 1.4	13.7 ± 1.4	13.7 ± 1.4	13.6 ± 1.4	13.7 ± 1.4
Sex, mean ± SD									
Female (n = 447)	30.5 ± 1.9	18.5 ± 1.9	11.0 ± 0.8	15.9 ± 1.4	14.0 ± 1.4	13.5 ± 1.4	13.5 ± 1.4	13.3 ± 1.4	13.5 ± 1.3
Male (n = 356)	32.5 ± 2.1	19.6 ± 2.0	11.8 ± 0.9	16.5 ± 1.5	14.3 ± 1.5	13.9 ± 1.4	14.0 ± 1.4	13.9 ± 1.4	14.0 ± 1.4
P-value	< 0.001	< 0.001	< 0.001	< 0.001	< 0.001	< 0.001	< 0.001	< 0.001	< 0.001
Race, mean ± SD									
Caucasian (n = 602)	31.7 ± 2.2	19.3 ± 2.1	11.4 ± 0.9	16.4 ± 1.5	14.3 ± 1.4	13.8 ± 1.4	13.9 ± 1.4	13.7 ± 1.4	13.8 ± 1.3
African American (n = 155)	30.6 ± 2.0	18.3 ± 1.8	11.0 ± 0.8	15.5 ± 1.4	13.5 ± 1.4	13.3 ± 1.4	13.4 ± 1.4	13.3 ± 1.4	13.4 ± 1.3
Others (n = 46)	30.2 ± 2.2	18.1 ± 1.9	10.9 ± 1.1	15.6 ± 1.6	13.6 ± 1.4	13.0 ± 1.5	13.1 ± 1.2	13.2 ± 1.3	13.4 ± 1.3
P-value	< 0.001^*^	< 0.001^*^	< 0.001^*^	< 0.001^*^	< 0.001^*^	< 0.001^*^	< 0.001^*^	< 0.05^*^	< 0.01^**^

The mean inner sagittal diameter of the atlas and the proportion of patients meeting atlas hypoplasia criteria, stratified by age group

When the ISD of the atlas was stratified by age (Table 2), there was no significant difference between groups (p > 0.05). There were also no significant differences in the rate of cases with atlas hypoplasia when comparing the same groups (p > 0.05).

**Table 2:** Inner sagittal diameter of the atlas and the rate of patients with atlas hypoplasia according to age groups The values of C1 ISD are given as the mean and standard deviation. P-values were calculated using an independent t-test for continuous variables, one-way ANOVA for variables with three or more degrees of freedom, and chi-squared test for categorical variables ISD: inner sagittal diameter; NS: not significant

Age	Number	C1 ISD (mm)	C1 hypoplasia ratio (%)
Overall	803	31.4 ± 2.2	2.1
< 30 years	205	31.3 ± 2.2	0.7
31-39 years	126	31.1 ± 2.1	0.4
40-49 years	118	31.4 ± 2.3	0.2
50-59 years	116	31.4 ± 1.9	0.1
60-69 years	64	31.8 ± 2.3	0
70-79 years	50	31.7 ± 2.5	0.2
80 ≤ years	124	31.4 ± 2.1	0.4
P	―	NS	NS

Correlation between each radiographic parameter

Pearson’s correlation analysis showed that the ISD of the atlas had a strong correlation with the SAC of the atlas (r = 0.86, p < 0.01); a moderate correlation with both the sagittal diameter of the dens (coefficient = 0.48, p < 0.01) and the ISD of C2 (r = 0.59, p < 0.01) to C3 (r = 0.44, p < 0.01); and a weak correlation with the ISD of C4 (r = 0.37, p < 0.01), C5 (r = 0.38, p < 0.01), C6 (r = 0.36, p < 0.01) and C7 (r = 0.37, p < 0.01). There was a moderate-to-strong correlation between each ISD below C2 (Table 3). No correlation was found between the sagittal diameter of the dens and the SAC of the atlas (r = 0.04, p > 0.05).

**Table 3:** Correlation matrix between each radiographic parameter ^**^P < 0.01 The values show the Pearson's correlation coefficient; p-values were also calculated using the Pearson's correlation coefficient equation ISD: inner sagittal diameter; SAC: space available for the cord; SD: sagittal diameter

Variable	C1 ISD	C1 SAC	Dens SD	C2 ISD	C3 ISD	C4 ISD	C5 ISD	C6 ISD	C7 ISD
C1 ISD	1	0.86^**^	0.48^**^	0.59^**^	0.44^**^	0.37^**^	0.38^**^	0.35^**^	0.37^**^
C1 SAC	0.86^**^	1	0.04	0.58^**^	0.42^**^	0.36^**^	0.35^**^	0.30^**^	0.30^**^
Dens SD	0.48^**^	0.04	1	0.17^**^	0.13^**^	0.10^**^	0.13^**^	0.14^**^	0.18^**^
C2 ISD	0.59^**^	0.58^**^	0.17^**^	1	0.86^**^	0.75^**^	0.67^**^	0.60^**^	0.57^**^
C3 ISD	0.44^**^	0.42^**^	0.13^**^	0.86^**^	1	0.88^**^	0.78^**^	0.67^**^	0.63^**^
C4 ISD	0.37^**^	0.36^**^	0.10^**^	0.75^**^	0.88^**^	1	0.89^**^	0.77^**^	0.67^**^
C5 ISD	0.38^**^	0.35^**^	0.13^**^	0.67^**^	0.78^**^	0.89^**^	1	0.86^**^	0.74^**^
C6 ISD	0.35^**^	0.30^**^	0.14^**^	0.60^**^	0.67^**^	0.77^**^	0.86^**^	1	0.85^**^
C7 ISD	0.37^**^	0.30^**^	0.18^**^	0.57^**^	0.63^**^	0.67^**^	0.74^**^	0.85^**^	1

Subaxial canal stenosis in atlas hypoplasia cases

A sub-cohort analysis of the 17 cases of atlas hypoplasia revealed that the mean ISD of the atlas was 26.3 ± 0.7 and 14.5 ± 0.8 mm, and the mean SAC of the atlas was 14.5 ± 0.8 mm within this cohort. The mean ISDs of the C2 to C7 levels were 13.9 ± 0.9, 12.2 ± 0.8, 12.0 ± 1.2, 12.0 ± 1.3, 12.1 ± 1.1, and 12.2 ± 1.0 mm, respectively, which were all smaller compared to the overall cohort mean ISDs. Within the hypoplasia group, the mean sagittal diameter of the dens was 10.7 ± 0.9 mm, which on average occupied 40.6 ± 0.0% of the C1 ISD. Regarding the rate of canal stenosis, the atlantal SAC was less than 14 mm in 17.6% of the cases, and the ISD of C2 to C7 were < 12 mm in 0%, 35.3%, 41.2%, 64.7%, 52.9%, and 47.1% of the cases, respectively (Table 4).

**Table 4:** Subaxial canal stenosis in atlas hypoplasia cases (n = 17) ^*^The values are given as the mean and standard deviation. ^**^Canal stenosis was defined as C1 SAC less than 14 mm and C2-C7 ISD less than 12 mm, respectively ISD: inner sagittal diameter; SAC: space available for the cord; SD: sagittal diameter

Variable	C1 ISD	C1 SAC	Dens SD	C2 ISD	C3 ISD	C4 ISD	C5 ISD	C6 ISD	C7 ISD
Diameter^*^ (mm)	26.3 ± 0.7	14.5 ± 0.8	10.7 ± 0.9	13.9 ± 0.9	12.2 ± 0.8	12.0 ± 1.2	12.0 ± 1.3	12.1 ± 1.1	12.2 ± 1.0
Canal stenosis ratio^**^ (%)	―	17.6	―	0	35.3	41.2	64.7	52.9	47.1

## Discussion

Atlas hypoplasia describes a malformation of the cervical arch during embryogenesis [12]. Historically, the posterior arch is the most frequent site of defect (3.4%) compared with the anterior arch (0.1%), which is consistent with findings of the current study [20]. The appearance of myelopathy associated with atlas dysplasia is extremely rare, primarily reported in anecdotal cases and small case series [7-11,13]. As a result, the pathophysiology of atlas hypoplasia is poorly understood, and the available literature does not establish a true causal relationship. For instance, both Yamahata et al. and Wang et al. have described a potential mechanistic relationship between atlas hypoplasia and atlantal stenosis, but neither was able to demonstrate a significant relationship within their studies [8,15].

Our current study is one of the first studies seen in the literature using a large, North American patient cohort. We found that cervical ISDs were generally larger in males versus females, and in Caucasians versus other races. There were also no significant relationships between age and ISD or stenosis ratios. Additionally, we did not find a strong correlation between atlantal (C1) ISD in relation to subaxial (C3-7) ISD, but we did see a strong inter-cervical (C3-7) relationship within the subaxial cervical spine. Within our cohort of cervical myelopathy patients, atlas hypoplasia was frequently accompanied by concurrent subaxial canal stenosis, a finding that is analytically distinct from the linear correlation analysis performed across the full cohort.

Demographic variability of sagittal diameter

Kelly et al. had previously reported a cadaveric study with 543 cervical spine specimens from people of Caucasian and African American descent. They measured the upper cervical sagittal diameters from the atlas, the axis, and C3 using digital calipers [14]. The ISD of the atlas ranged from 23.5 to 38.1 mm, with a mean of 30.8 ± 2.4 mm, and defined atlas hypoplasia as an ISD ≤ 26.1 mm within their cohort, which fell in the lowest 2.5% of measurements (two standard deviations below the mean). They also found that Caucasian specimen ISDs were slightly larger compared to their African American counterparts (Caucasians: 31.3 ± 2.3 mm vs. African Americans: 30.3 ± 2.2 mm). These values are similar to the results of the current study, validating our use of CT measurements for sagittal diameter.

Yamahata et al. performed a similar CT-based study, but reported a mean atlantal ISD of 29.7 ± 2.0 mm in a Japanese cohort with 102 patients [15]. The lower value may be attributed to higher rates of atlantal stenosis associated with atlas hypoplasia in East Asian populations, leading to a smaller diameter, as well as generally smaller individuals due to dietary and lifestyle differences [13]. Regarding the subaxial cervical spinal canal, Lee et al. evaluated sagittal diameters from the C3 to C7 vertebrae in 469 adult skeletal specimens from Caucasian and African American patients. They reported an average ISD of 14.1 ± 1.6 mm, which was slightly larger than in the current study [3]. 

Age and canal stenosis

Lee et al. also estimated that the prevalence of bony cervical spinal stenosis was 4.9% in adults, 6.8% in those older than 50 years, and 9% in those older than 70, adopting a similar threshold of a canal diameter of < 12 mm as stenotic [3]. Similarly, Yukawa et al. reported that the ISD of C2 to C7 had an inverse relationship with age in 1,200 asymptomatic Japanese individuals [18]. Although subaxial stenosis is known to correlate with increasing age, neither the ISD of the atlas nor the prevalence of atlas hypoplasia was associated with age in the present study. The atlas ring does not seem to become stenotic secondary to age-related degenerative changes, indicating that atlas hypoplasia should be regarded as a congenital rather than developmental pathology.

Atlas hypoplasia and canal stenosis

Although the correlation between the ISD of the atlas and subaxial ISD was weak to moderate within our cohort of cervical myelopathy patients, atlas hypoplasia was frequently accompanied by concurrent subaxial canal stenosis. These represent distinct findings, as a weak linear correlation does not preclude a high prevalence of stenosis within the hypoplastic subgroup. In fact, roughly one in five patients with atlas hypoplasia fit the criteria for stenosis. This aligns with findings of the study by Yamahata et al., where patients with symptomatic atlas hypoplasia had concomitant subaxial stenosis represented by a mean C4 ISD of 11.9 ± 0.8 mm. Conversely, when viewing patients with symptomatic subaxial stenosis, they often had a normal atlantal ISD [15,21]. 

It is also unclear if atlantal hypoplasia independently causes symptomatic cervical myelopathy or radiculopathy. Several articles have reported that very few patients develop symptomatic myelopathy from compression of the atlas ring without other concomitant pathologies, such as transverse ligament ossification, retro-odontoid pseudotumor, and atlantoaxial subluxation [9,13,19]. This may explain why the development of myelopathy due to atlas-level stenosis is more common in older adults, as the prevalence of these secondary conditions correlates with age. With this in mind, screening for atlantal stenosis may be helpful in stratifying patients who are at heightened risk for cervical myelopathy. Oshima et al. had previously described a C2/C3 spinolaminar test using lateral radiographs to look at atlantal SAC to check for stenosis; if this returned positive, then a follow-up MRI should be performed [22]. 

Impact of dens size

Wang et al. and Kelly et al. identified hypertrophy of the dens as a related etiology for atlantal stenosis [8,14]. In the present study, the mean occupancy rate of the dens in the ISD of the atlas in atlas hypoplasia cases exceeded 40%, suggesting that this condition may also contribute to the development of myelopathy. Variation in dens size and morphology with age may be worthwhile to investigate in order to better understand the causes of axial myelopathies.

Strengths and limitations

In this study, we presented the largest sample size to date to examine the association between atlas hypoplasia, aging, and subaxial stenosis. There are several limitations, as with any study. The retrospective design using a homogeneous cohort may affect generalizability and result in selection bias. The study also did not include patients with associated genetic disorders that could cause atlas-level stenosis, such as os odontoideum and Klippel-Feil syndrome. Additional research into predisposing pathologies may be warranted. In addition, this study did not evaluate soft tissue damage, spinal cord injury, or atlantoaxial instability. Further analysis of the degree of cord compression and factors associated with stenosis, along with atlas hypoplasia, would be useful. The atlas hypoplasia subgroup was small, with only 17 patients, which limits the statistical strength of conclusions drawn from this subgroup analysis and should be interpreted with caution.

Furthermore, while CT measurements were performed by fellows under the supervision of orthopaedic spine surgeons with experience in cervical radiographic anatomy, formal intra-rater and inter-rater reliability testing was not conducted, which represents an additional methodological limitation. Finally, this study was intentionally designed as a radiographic and anatomical investigation and did not include correlation with clinical symptoms, neurologic examination findings, MRI-based cord signal changes, or long-term patient outcomes. Such correlations would substantially strengthen the clinical relevance of these findings and are identified as a priority area for future investigation.

## Conclusions

In our cohort, the prevalence of atlas hypoplasia was not associated with older age, differing from subaxial developmental canal stenosis. Furthermore, there was no strong correlation between the ISD of the atlas and each subaxial ISD. However, within the atlas hypoplasia subgroup, a notable proportion of patients met the criteria for subaxial canal stenosis. While there are multiple avenues for future research, we believe that this study provides a more refined pathogenetic understanding of this uncommon condition.
